# Reimbursement restrictions reduce liver fibrosis screening in adults with diabetes in Ontario: a population-based interrupted time series analysis

**DOI:** 10.1038/s41598-026-48600-5

**Published:** 2026-04-29

**Authors:** Jessica Burnside, Maya Djerboua, Jennifer A. Flemming, Giada Sebastiani, William W. L. Wong, Zihang Lu, Harpreet S. Bajaj, Keyur Patel, Mia Biondi, Michael Betel, Amy Nahwegahbow, Zoë R. Greenwald, Erica E. M. Moodie, Sahar Saeed

**Affiliations:** 1 Department of Public Health Sciences, Queen’s University, 203 Carruthers Hall, 62 Fifth Field Company Lane, Kingston, ON K7L 3N6 Canada; 2 ICES Queen’s, Kingston, ON Canada; 3 Division of Gastroenterology and Hepatology, Department of Medicine, McGill University Health Centre, Montreal, QC Canada; 4 School of Pharmacy, University of Waterloo, Kitchener, ON Canada; 5 LMC Healthcare, Brampton, ON Canada; 6 Division of Gastroenterology and Hepatology, University Health Network Toronto, Toronto, ON Canada; 7 School of Nursing, York University, Toronto, ON Canada; 8 Fatty Liver Alliance, Toronto, ON Canada; 9 School of Public Health Sciences, University of Waterloo, Kitchener, Canada; 10 Dalla Lana School of Public Health, University of Toronto, Toronto, ON Canada; 11 Department of Epidemiology & Biostatistics, McGill University, Montreal, QC Canada

**Keywords:** Liver fibrosis, Screening, MASLD, Diabetes complications, Healthcare quality, Diseases, Endocrinology, Gastroenterology, Health care, Medical research, Risk factors

## Abstract

Adults with type 2 diabetes have increased risk of liver fibrosis, leading guidelines to recommend screening with the Fibrosis-4 Index (FIB-4). We assessed if modifications to Ontario Health Insurance Plan coverage of aspartate aminotransferase (AST) tests in 2013 were associated with changes in liver fibrosis assessment rates among adults with diabetes. This population-based cohort study included adults with diabetes and no history of liver disease in Ontario, Canada, from 2010 to 2022. Policy periods were constructed based on AST reimbursement practices. The primary outcome was the receipt of all laboratory tests needed to calculate FIB-4 (AST, alanine aminotransferase, and platelet count). Modified Poisson regression estimated the adjusted risk ratios (aRRs) for predictors of receiving liver fibrosis assessment. Interrupted time series analysis employed autoregressive integrated moving average models to assess both immediate and sustained policy effects. This study included 1,680,451 individuals; 47% were female; median age was 64 years [IQR, 55–73 years]. The annual proportion without any testing ranged from 39 to 47%. The risk of receiving all required tests for FIB-4 declined significantly: 12.7% (aRR 0.873, 0.869–0.877) during implementation, 33.9% (aRR 0.661, 0.657–0.664) post-intervention, and 63.1% (aRR 0.369, 0.366–0.371) COVID-19. Interrupted time-series analysis showed a significant immediate decline in monthly testing of FIB-4 components (− 0.38; 95% CI − 0.59 to − 0.18), followed by a continued monthly decrease (− 0.03; 95% CI − 0.07 to 0.01). Restricting AST reimbursement was associated with a decline in liver fibrosis assessments for people with diabetes despite increasing consensus in favor of preventative screening.

**Supplementary Information:**

The online version contains supplementary material available at 10.1038/s41598-026-48600-5.

## Introduction

Chronic liver disease is now one of the leading causes of morbidity and mortality worldwide, primarily driven by the rising prevalence of metabolic dysfunction-associated steatotic liver disease (MASLD), which affects an estimated 38% of the global population^1^. Progression from steatosis to liver fibrosis^2^ is a key predictor of adverse outcomes, including cardiovascular disease, end-stage liver disease, and all-cause mortality^3,4^. Individuals with type 2 diabetes are at high risk of liver fibrosis progression^2^. Accordingly, since 2016, leading liver, obesity, and diabetes associations in Europe, the United States, and Latin America have recommended screening for advanced liver fibrosis in this population^5–9^. Several clinical risk-stratification pathways have been proposed, with the most widely adopted approach utilizing the Fibrosis-4 Index (FIB-4), which requires aspartate aminotransferase (AST), alanine aminotransferase (ALT), platelet count and age for its calculation. Most recently, Diabetes Canada released the first Canadian clinical practice guidelines to include advanced fibrosis screening with FIB-4 for people with type 2 diabetes, emphasizing the growing importance of this tool in practice^10^.

Although core health services in Canada are publicly funded, provincial and territorial governments have substantial authority over how services are organized, delivered, and managed. Laboratory testing is the highest-volume medical activity in healthcare^11^. In 2012, Ontario, Canada’s most populous province, introduced an *Appropriateness Initiative* to assess health interventions that might be misused^12^. The goal was to reduce inappropriate or unnecessary care stemming from both underutilization (when needed services are not provided) and overutilization (when services are given without clinical need)^11^.

The first phase of the *Appropriateness Initiative* evaluated the clinical utility of six blood tests, including aspartate aminotransferase (AST)^13^. In January 2013, the Ontario Health Technology Advisory Committee recommended that the Ontario Health Insurance Plan (OHIP) restrict AST reimbursement to cases ordered on the advice of physicians specializing in liver disorders^13–15^. The policy rationale emphasized the limited clinical utility of AST in community-based laboratories. AST is found in several organs such as the heart, liver and muscle making it less specific for liver disease than ALT, which remained unrestricted^13^. Since primary care delivers approximately 80% of diabetes care in Canada^16^, the policy effectively curtailed access to AST testing in primary care settings^17^.

AST is an essential component of the FIB-4 index, such that the policy created a structural barrier to evidence-based screening. In this study, we assessed trends in liver function testing and evaluated the impact of Ontario’s reimbursement policy change on the assessment of fibrosis risk among individuals with diabetes. The implications of this natural experiment extend beyond Ontario, offering critical insights for jurisdictions worldwide as they balance cost-containment measures with the need to maintain equitable access to evidence-based chronic disease prevention.

## Methods

### Data source

We conducted a retrospective population-based cohort study using administrative health data from ICES, an independent, non-profit research institute authorized under Ontario’s health information privacy laws to use health data for system evaluation and improvement. ICES databases contain individual-level data on healthcare encounters for all residents eligible for the Ontario Health Insurance Plans (OHIP)^18^. Fourteen data holdings from ICES were utilized in this study (Supplemental 1). These datasets were linked using unique encoded identifiers.

### Eligibility criteria

Individuals with diabetes were identified using the Ontario Diabetes Dataset, which employs a validated algorithm based on two physician service claims for diabetes within two years or one hospitalization with a diagnosis of diabetes^19^, estimated to have 89.3% sensitivity and 97.6% specificity^20^. Individuals with diabetes aged 40 years and older between January 1, 2010, and June 30, 2022, were included in the study. The age of 40 was selected to align with guidelines that recommend routine screening for diabetes^21^. We excluded individuals with invalid sex, date of birth, date of death, non-Ontario residency, prevalent liver diseases (Supplemental 2), or no history of routine laboratory tests before study entry (Supplemental 3). Individuals were censored when an incident of liver disease occurred or at the end of the follow-up or at death. Periods of time while individuals were hospitalized or pregnant were also excluded to reflect routine testing patterns.

### Exposure: policy period

We divided calendar time into four periods: Pre-intervention (January 2010–December 2012): No restrictions on ordering AST. Implementation (January 2013–March 2017): The Ontario Ministry of Health communicated the restriction to providers via InfoBulletins in January and April 2013. Notices from laboratories informing providers how to document test eligibility for insured services were released up to March 2017^22^. Post-intervention (April 2017–February 2020): Consistent application and communication of eligibility for OHIP-covered AST^15^. COVID-19 Pandemic (March 2020–June 2022): Broad disruptions to healthcare services.

### Outcome: liver testing modalities

Logical observation identifiers names and codes (LOINCs) were used to identify AST, ALT and platelets tests (Supplemental 3). Three liver testing modalities were evaluated hierarchically based on the clinical utility of identifying liver fibrosis. Optimal testing included being tested by all the components needed to calculate a FIB-4 (AST, ALT and platelets), which were ordered on the same date. FIB-4 is guideline recommended for fibrosis risk stratification^5–10^. To capture attempted assessment of fibrosis risk using less accurate AST-based methods, for example the AST to ALT ratio^23,24^, we quantified when ALT and AST were ordered within 6 months. Finally, as the least appropriate method to identify fibrosis risk, we assessed when ALT was ordered alone^25^. In practicality, however, this is the most used laboratory measure to assess general liver injury in primary care^26^. If multiple testing modalities were identified during the period of interest, the individual was classified based on a hierarchical approach.

### Covariates

Sociodemographic variables analyzed included sex, age, and rurality, as well as area level proxies for socioeconomic status. History of alcohol and substance use disorder and hypertension, hemoglobin A1c (HbA1c), duration and type of diabetes, as well as aggregated diagnosis groups, were the clinical attributes assessed. Sex-specific upper limits of normal were used for ALT: 35 U/L (male) and 25 U/L (female)^27^. Roster status with a primary care provider (PCP) and resource utilization bands were also considered. Algorithms and definitions are available in Supplemental 4.

### Statistical analysis

Descriptive statistics included the annual proportion of the cohort tested by each modality (April to March) and by policy era. A one-year lookback period was used to identify individuals considered “at-risk” for testing. Specifically, if an individual was tested, they were not considered “at risk” for testing modalities of equal or lesser hierarchical rank for 12 months (Supplemental 5).

To assess predictors of laboratory testing, we used a modified Poisson regression with generalized estimating equations to account for repeated measures. Adjusted models included: sex, age, income quintile, diabetes duration, HbA1c, hypertension, PCP status, rurality and policy era. All covariates were updated at the beginning of each policy period. A complete case analysis was performed.

For the interrupted time series (ITS) analysis, we applied an Autoregressive Integrated Moving Average (ARIMA) model to estimate the immediate effects (step) and gradual changes (ramp) of the policy periods on the monthly testing rate per 100 people at risk. Forecasted testing rates in the absence of policy changes were also ascertained from the ARIMA model. First-order and yearly seasonal differencing were applied to achieve stationarity^28^. Model parameters were selected using the Box-Jenkins method, guided by autocorrelation and partial autocorrelation functions, and optimized using the Akaike Information Criterion.

All data were prepared and analyzed using SAS Enterprise Guide, Version 7.1 (SAS Institute Inc., Cary, North Carolina, USA).

### Ethics

ICES manages a large repository of population-level health data and operates under a strict legal and ethical framework. As a designated “prescribed entity” under Ontario’s Personal Health Information Protection Act (PHIPA), ICES is authorized to collect personal health information, without consent, for the purpose of analysis or compiling statistical information with respect to the management of, evaluation or monitoring of, the allocation of resources to or planning for all or part of the health system. The use of the data in this project is authorized under Sect. 45 of PHIPA and approved by ICES’ Privacy and Legal Office. The study was approved by the Queen’s University Health Sciences and Affiliated Teaching Hospitals Research Ethics Board (#6040592) and waived informed consent because deidentified data were used.

## Results

**Fig. 1:**
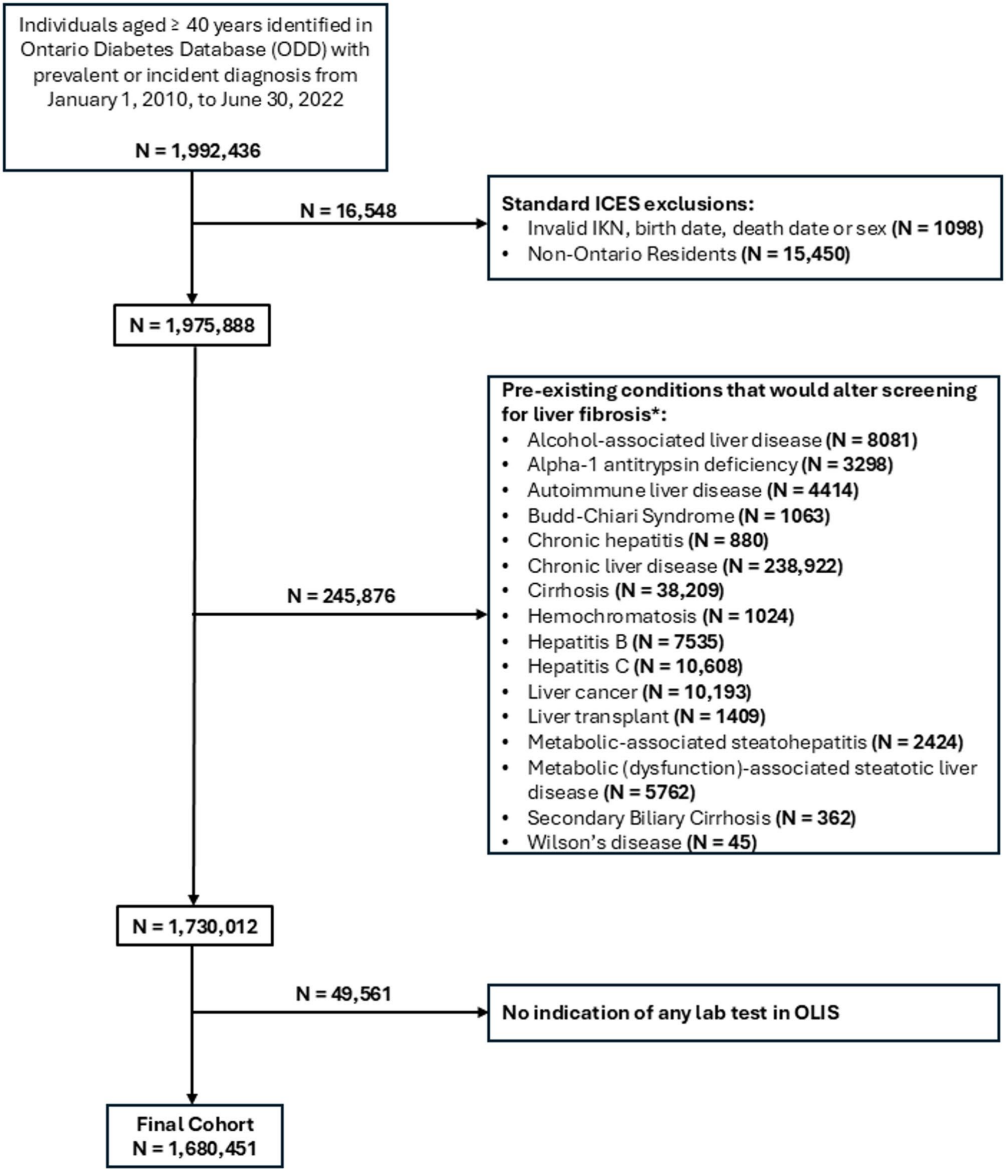
Cohort Creation Flowchart Flowchart with top box indicating the number of individuals identified based on inclusion criteria. Read top to bottom to see the number of individuals excluded. The final cohort size is 1,680,451. *The ‘N’ values for each liver condition listed are not mutually exclusive.

We identified 1,992,436 people 40 or older with diabetes. After exclusions of invalid data or non-Ontario residency (n = 16,548), prevalent liver conditions (n = 245,876), or lack of laboratory records (n = 49,561), a total of 1,680,451 individuals were included (Fig. 1). This corresponded to 4,726,622 person-years with a median follow-up time of 7.6 years (interquartile range [IQR]: 3.4–12.5) per individual. The cohort consisted of 47% females, median age 64 years (IQR: 55–73), 66% had hypertension, and 88% were high or very high users of the health system (Table 1). The characteristics of the cohort remained similar across policy eras.

**Table 1:** Sociodemographic and clinical characteristics of the study cohort overall, and by policy period, based on person-years (py).

Sample Characteristic	Overall (4,726,622 py)	Pre-Intervention (1,011,023 py)	Implementation (1,199,643 py)	Post-Intervention (1,237, 627 py)	COVID-19 Pandemic (1,278,329 py)
*Sex*					
Female	47% (2,242,839)	48% (480,662)	48% (570,815)	47% (586,328)	47% (605,034)
*Age (years)*					
40–49	14% (673,705)	16% (162,317)	16% (188,646)	13% (165,788)	12% (156,954)
50–59	23% (1,087,326)	24% (238,125)	24% (286,141)	23% (284,595)	22% (278,465)
60–69	28% (1,318,397)	27% (275,151)	28% (334,320)	28% (350,670)	28% (358,256)
70–79	22% (1,036,821)	21% (212,258)	20% (243,496)	22% (274,167)	24% (306,900)
80 +	13% (610, 373)	12% (123,172)	12% (147,040)	13% (162,407)	14% (177,754)
*Rurality*					
Urban	88% (4,158,816)	88% (885,718)	88% (1,053,421)	88% (1,092,359)	88% (1,127,318)
Rural	11% (534,754)	12% (124,735)	12% (142,307)	11% (134,134)	10% (133,578)
Missing	0.7% (33,052)	0.06% (570)	0.3% (3,915)	0.9% (11,134)	1.4% (17,433)
*Income quintile*					
1 – least well off	22% (1,054,706)	22% (220,130)	22% (260,069)	23% (282,402)	23% (292,105)
2	21% (1,012,993)	21% (216,018)	21% (255,646)	22% (267,462)	21% (273,867)
3	20% (956,825)	20% (204,703)	20% (244,029)	20% (251,736)	20% (256,357)
4	19% (888,450)	19% (196,874)	20% (233,822)	18% (225,308)	18% (232,446)
5 – most well off	16% (770,296)	17% (168,121)	16% (197,485)	16% (199,024)	16% (205,666)
Missing	0.9% (43,454)	0.5% (5177)	0.7% (8592)	1% (11,695)	1.4% (17,888)
*Age and labor force quintile*					
1 – least marginalized	21% (990,655)	19% (193,155)	21% (253,146)	21% (264,991)	22% (279,363)
2	19% (874,905)	18% (184,650)	18% (220,971)	19% (233,072)	18% (236,212)
3	18% (834,991)	18% (186,223)	18% (214,389)	17% (214,265)	17% (220,114)
4	18% (850,776)	19% (189,005)	18% (216,310)	18% (221,014)	18% (224,447)
5 – most marginalized	23% (1,101,937)	25% (248,557)	23% (280,221)	23% (282,080)	23% (291,079)
Missing	1.6% (73,358)	0.9% (9,433)	1.2% (14,606)	1.8% (22,205)	2.1% (27,114)
*Material resources quintile*					
1 – least marginalized	16% (744,145)	15% (151,923)	15% (183,213)	17% (212,135)	15% (196,874)
2	18% (864,981)	17% (172,945)	17% (209,141)	19% (234,869)	19% (248,026)
3	20% (934,019)	19% (193,765)	19% (231,640)	20% (244,280)	21% (264,334)
4	21% (1,006,447)	22% (225,753)	22% (266,893)	21% (254,668)	20% (259,133)
5 – most marginalized	23% (1,103,672)	25% (257,204)	25% (294,150)	22% (269,470)	22% (282,848)
Missing	1.6% (73,358)	0.9% (9,433)	1.2% (14,606)	1.8% (22,205)	2.1% (27,114)
*Racialized and newcomer populations quintile*					
1 – least marginalized	17% (804,300)	18% (181,359)	17% (206,589)	17% (213,162)	16% (203,190)
2	17% (790,988)	18% (179,227)	17% (203,373)	16% (202,857)	16% (205,531)
3	16% (770,504)	17% (169,610)	16% (196,033)	16% (197,540)	16% (207,321)
4	19% (893,463)	19% (188,731)	19% (223,833)	19% (229,610)	20% (251,289)
5 – most marginalized	29% (1,394,009)	28% (282,663)	30% (355,209)	30% (372,253)	30% (383,884)
Missing	1.6% (73,358)	0.9% (9,433)	1.2% (14,606)	1.8% (22,205)	2.1% (27,114)
*Household and dwellings quintile*					
1 – least marginalized	20% (963,833)	20% (198,707)	20% (245,647)	20% (249,778)	21% (269,701)
2	18% (834,362)	18% (179,572)	18% (212,695)	18% (221,274)	17% (220,821)
3	18% (851,120)	18% (184,863)	18% (216,161)	18% (222,887)	18% (227,209)
4	19% (908,513)	20% (201,524)	19% (232,204)	19% (232,056)	19% (242,729)
5 – most marginalized	23% (1,095,436)	23% (236,924)	23% (278,330)	23% (289,427)	23% (290,755)
Missing	1.6% (73,358)	0.9% (9,433)	1.2% (14,606)	1.8% (22,205)	2.1% (27,114)
*Diabetes duration*					
< 2 years	9% (424,170)	9% (94,554)	9% (111,653)	8% (104,771)	9% (113,192)
2–5 years	21% (1,002,258)	26% (261,224)	21% (248,290)	20% (251,777)	19% (240,967)
6–10 years	24% (1,140,679)	28% (283,311)	26% (307,689)	22% (277,349)	21% (272,330)
11–15 years	20% (939,171)	18% (181,408)	21% (245,933)	21% (261,090)	20% (250,740)
16–20 years	14% (648,904)	13% (132,442)	12% (148,067)	14% (171,360)	15% (197,035)
20 + years	12% (571,440)	6% (58,084)	12% (138,011)	14% (171,280)	16% (204,065)
*HbA1c*					
< 7.0%	42% (1,980,715)	38% (380,177)	42% (498,366)	45% (560,778)	42% (541,394)
7.0–8.5%	23% (1,066,675)	21% (214,297)	22% (269,567)	24% (291,797)	23% (291,014)
> 8.5%	10% (452,690)	9% (93,405)	10% (120,733)	10% (123,445)	9% (115,107)
Missing	26% (1,226,542)	32% (323,144)	26% (310,977)	21% (261,607)	26% (330,814)
*Hypertension*					
Yes	66% (3,111,214)	66% (668,719)	65% (784,347)	66% (816,068)	66% (842,080)
*Primary Care Provider Status*					
Not Rostered	3% (135,000)	2% (18,491)	2% (28,758)	3% (38,731)	4% (49,020)
Rostered	83% (3,913,959)	81% (822,188)	83% (992,716)	84% (1,036,164)	83% (1,062,891)
Virtually Rostered	14% (677,663)	17% (170,344)	15% (178,169)	13% (162,732)	13% (166,418)
*Alcohol use disorder*					
Yes	4% (209,239)	5% (48,142)	5% (54,268)	4% (53,065)	4% (53,764)
*Substance use disorder*					
Yes	7% (335,664)	7% (69,851)	7% (85,170)	7% (88,694)	7% (91,949)
Number of major Aggregated Diagnosis Groups	3 [2–4]	3 [2–4]	3 [2–4]	3 [2–4]	3 [1, 4]
*Resource Utilization Band*					
Non-users	1% (48,514)	0.4% (4092)	0.9% (10,915)	1% (15,162)	1% (18,345)
Healthy Users	0.22% (10,196)	0.1% (1,089)	0.2% (2,346)	0.2% (3,133)	0.3% (3,628)
Low Morbidity	1.1% (53,330)	0.6% (5,998)	1% (12,304)	1% (16,079)	1% (18,949)
Moderate	9.5% (450,531)	8% (80,672)	9% (110,378)	10% (124,983)	11% (134,498)
High	35% (1,644,868)	31% (314,723)	34% (405,708)	36% (447,250)	37% (477,187)
Very High	53% (2,519,183)	60% (604,449)	55% (657,992)	51% (631,020)	49% (625,722)
ALT^a^	24 [18–33]	23 [18–32]	24 [18–33]	24 [18–34]	24 [18–34]
Missing	46% (2,153,543)	50% (501,333)	46% (556,636)	44% (547,266)	43% (548,308)
AST^a^	22 [18–28]	22 [19–28]	22 [18–28]	22 [18–28]	22 [18–28]
Missing	81% (3,818,420)	79% (795,479)	80% (958,399)	81% (1,005,080)	83% (1,059,462)
Platelets^a^	237 [199–280]	232 [194–276]	235 [198–279]	238 [201–281]	240 [203–284]
Proportion with platelet testing in 1-year lookback	59% (2,783,894)	54% (548,965)	58% (692,638)	60% (747,170)	62% (795,121)
Missing	41% (1,942,728)	46% (462,058)	42% (507,005)	40% (490,457)	38% (483,208)
Total cholesterol : HDL-C ratio^a^	4 [3–5]	4 [3–5]	4 [3–5]	4 [3–5]	4 [3–5]
LDL-C (mmol)^a^	2 [2, 3]	2 [2, 3]	2 [2, 3]	2 [2, 3]	3 [2, 3]

^a^Most recent with 1 year lookback. Categorical variables are presented as % (n), while continuous variables include the median [interquartile range]. [Abbreviations: aspartate aminotransferase (AST), alanine aminotransferase (ALT) primary care provider (PCP), hemoglobin A1c (HbA1c), high-density lipoprotein (HDL), and low-density lipoprotein (LDL)].

### Annual proportion screened

**Fig. 2:**
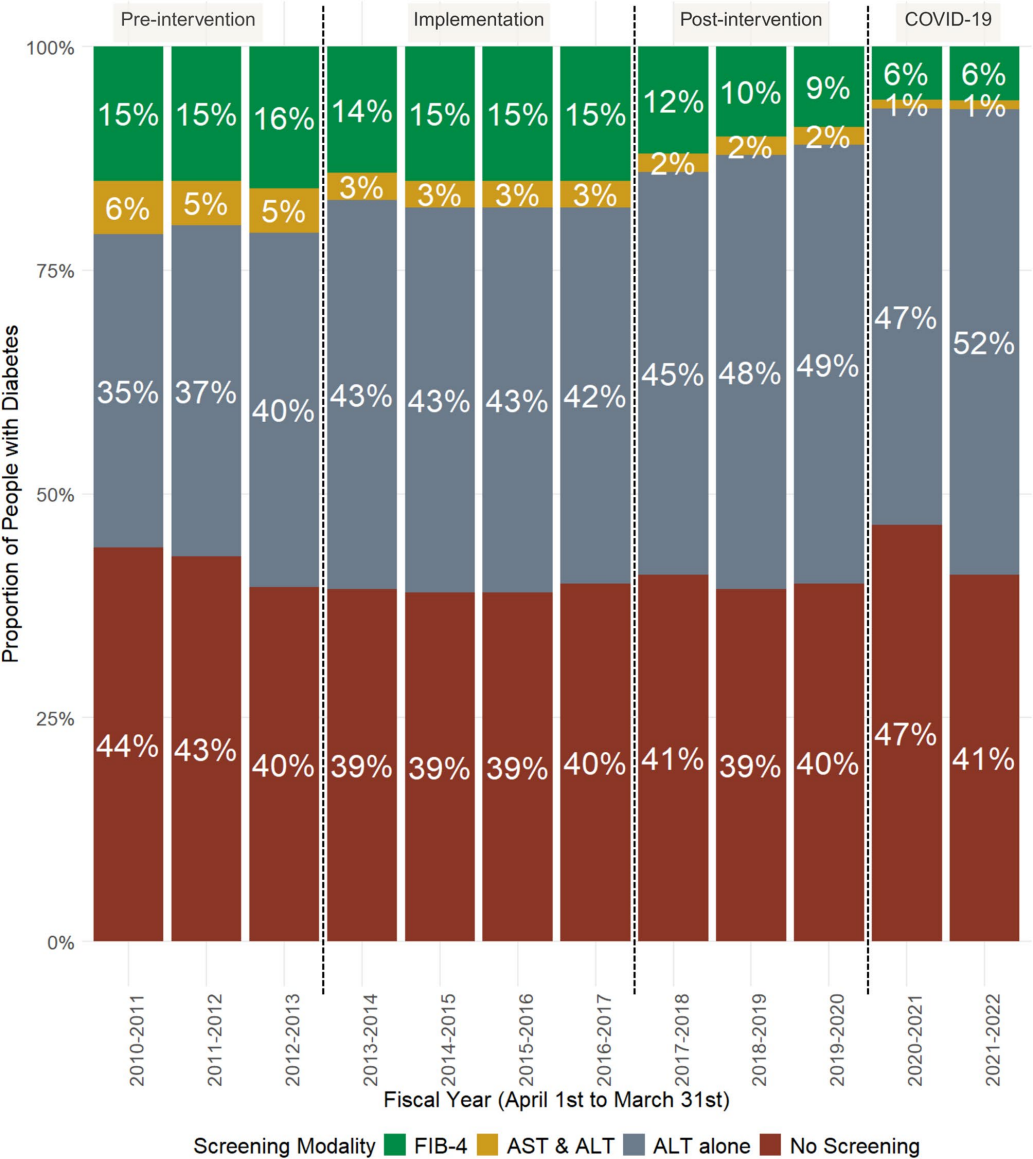
Annual liver testing modalities among people with diabetes. Stacked bar chart with the proportion of people with diabetes who received FIB-4 (green), ALT plus AST (yellow), ALT alone (grey) and no screening (red) modalities per fiscal year. White values within each bar depict exact values. Approximate policy periods are delineated through the use of black vertical lines and beige labels at the top of the chart.

Between 2010 and 2021, the annual proportion of the cohort without any liver testing remained constant (44–41%), with a peak at the onset of the COVID-19 pandemic (47%) (Fig. 2). Shifts among other testing modalities were observed: ALT-alone testing increased from 35% (n = 314,666) to 51% (n = 623,821); ALT plus AST testing decreased from 6% (n = 50,720) to 1% (n = 12,528); and components for FIB-4 testing dropped from 15% (n = 129,510) to 6% (n = 71,153). The trends observed were consistent with the proportion tested throughout the policy eras (Supplemental 6). Throughout the study period, FIB-4 could not be computed for more than half of individuals (55%, n = 929,443).

### Cascade of care

Among people who received an ALT test, 20% (n = 2,279,673) had results above the upper limit of normal, and only 15% (n = 349,263) were followed up with additional testing with either AST or the components to calculate FIB-4 testing within a year.

### Factors associated with testing modality

#### Policy-level

**Fig. 3:**
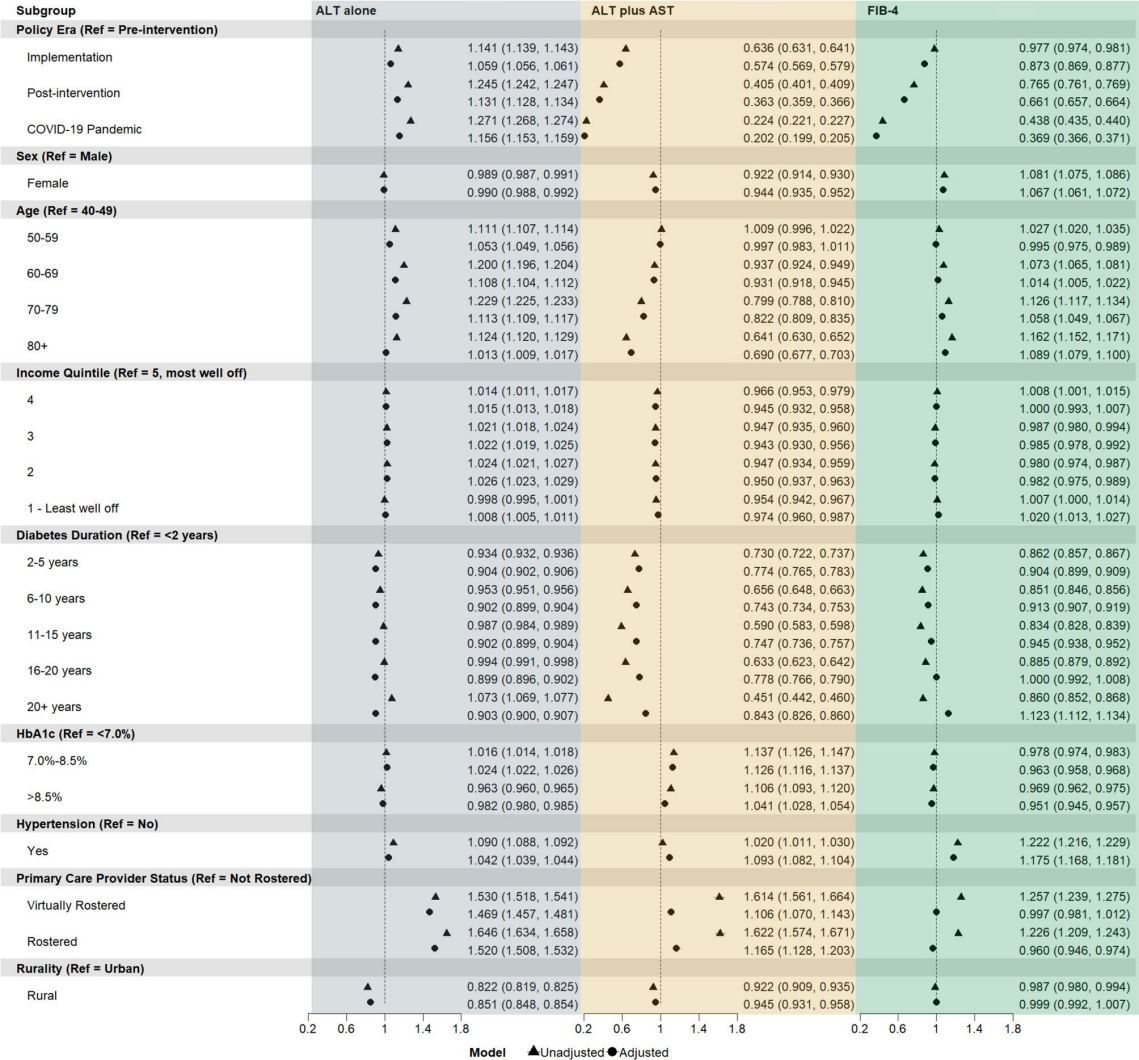
Factors associated with liver testing modality. Forest plot depicting the unadjusted (triangle) and adjusted (circle) relative risk with 95% confidence intervals for 3 separate modified Poisson regressions for i) ALT alone (grey), ii) ALT plus AST (yellow), and iii) FIB-4 (green) from January 2010 to June 2022. In the visual of the point estimates, the associated confidence intervals are often so small that they are not visible. Note, ‘Ref’ is being used as a short form for Reference.

Relative to the pre-intervention period, there was a monotonic increase in ALT testing over the various intervention periods, starting with the implementation (adjusted relative risk [aRR] 1.059, 95%CI 1.056–1.061), followed by the post-intervention (aRR 1.131, 1.128–1.134) and peaking during COVID-19 (aRR 1.156, 1.153–1.159) (Fig. 3). The policy era was the strongest predictor of AST-based liver testing modalities. The likelihood of ALT plus AST testing decreased inversely to ALT testing [(aRR 0.574, 0.569–0.579) during implementation, (aRR 0.363, 0.359–0.366) during post-intervention, and (aRR 0.202, 0.199–0.205) during the pandemic]. FIB-4 testing decreased, but to a lesser extent than ALT plus AST. Specifically, by 12.7% (aRR 0.873, 0.869–0.877) during implementation, 33.9% (aRR 0.661, 0.657–0.664) post-intervention, and 63.1% (aRR 0.369, 0.366–0.371) the COVID-19 period.

#### Provider-level

Individuals rostered to a PCP, compared to those not attached, had a 52.0% (aRR 1.520, 1.508–1.532) higher likelihood of ALT testing. Similarly, evidence of being registered with a PCP corresponded to a 16.5% (aRR 1.165, 1.128–1.203) increase in the risk of ALT plus AST testing. This relationship was not observed for testing FIB-4 components; those rostered with primary care were 4% (aRR 0.960, 0.946–0.974) less likely to be tested.

#### Patient-level

Females were 6.7% (aRR 1.067, 1.061–1.072) more likely to be tested with all FIB-4 components. Hypertension was associated with an increased risk of all types of testing: 4.2% (aRR 1.042, 1.039–1.044) for ALT alone, 9.3% (aRR 1.093, 1.082–1.104) for ALT plus AST, and 17.5% (aRR 1.175, 1.168–1.181) for FIB-4, compared to those without hypertension. Individuals with HbA1c levels of 7–8.5% or > 8.5%, were associated with a 3.7% (aRR 0.963, 0.958–0.968) and 4.9% (aRR 0.951, 0.945–0.957) decreased risk of FIB-4 component testing, respectively, compared to HbA1c levels of < 7%. In contrast, the 7–8.5% and > 8.5% HbA1c groups had 12.6% (aRR 1.126, 1.116–1.137) and 4.1% (aRR 1.041, 1.028–1.054) increased risk of ALT plus AST testing, respectively. Living in a rural area decreased the risk of testing with ALT alone by 14.9% (aRR 0.851, 0.848–0.854) and ALT plus AST by 5.5% (aRR 0.945, 0.931–0.958) compared to urban dwellers but did not significantly affect FIB-4 component testing. The impact of area-level income quintile was minimal across all screening types.

### Impact of the policy on absolute monthly rates

#### FIB-4

**Fig. 4:**
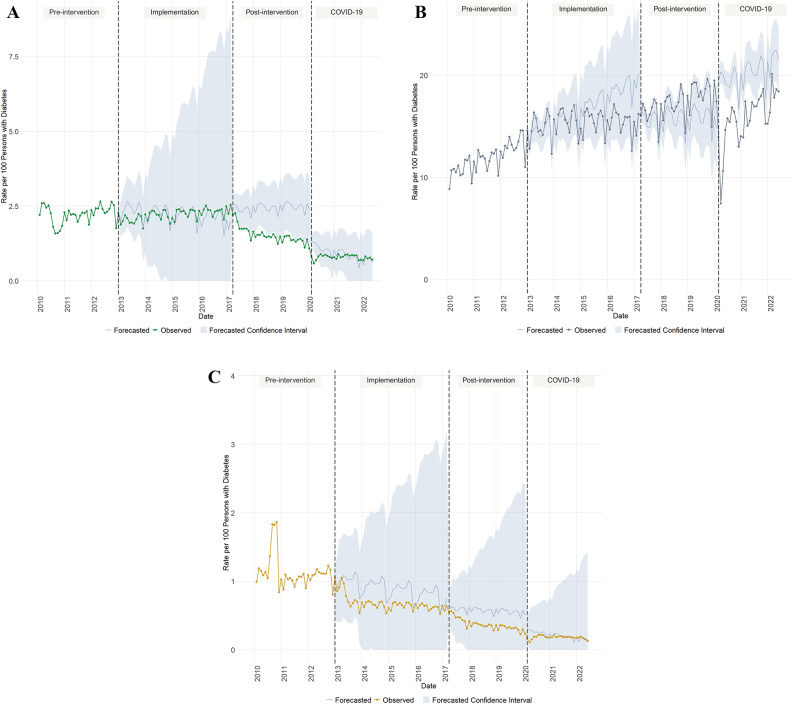
Impact of the policy periods on absolute testing rates. Line graphs show the observed monthly rate of testing with (**A**) FIB-4 (green), (**B**) ALT (grey), and (**C**) ALT plus AST (yellow). Forecasted rates calculated with the ARIMA models are depicted in light blue with shaded confidence intervals (cut-off at zero). Policy periods are delineated through the use of black vertical lines and beige labels at the top of the graph.

Nearly identical median monthly rates of FIB-4 testing were observed during pre-intervention (2.27, IQR: 2.14–2.42) and implementation (2.24, IQR: 2.07–2.35) and no statistically significant step or ramp changes were noted (Fig. 4A). In the post-intervention period, there was a significant immediate decline (step: −0.38, 95% CI −0.59, −0.18) paired with an additional decline of −0.03 (95%CI −0.07, 0.01) tests per month (Supplemental 7). During COVID-19, FIB-4 testing experienced a further immediate decrease (step: −0.70, 95% CI −1.00, −0.41), followed by a transient rebound (pulse: 0.25, 95% CI 0.04, 0.46), but no sustained ramp change over time. These shifts were reflected in the lower median monthly testing rate in post-intervention (1.49 per 100 persons, IQR: 1.38–1.64) and COVID-19 (0.81, IQR: 0.74–0.85).

#### ALT alone

During the pre-intervention period, the median monthly rate of testing with ALT alone was 11.9 (IQR: 10.7–12.7) per 100 persons. The rate of testing with ALT stagnated and then increased in the implementation and post-intervention periods compared to forecasts (Fig. 4B), but no significant changes in step or ramp were noted. The median monthly testing rates were 15.7 (IQR: 14.5–16.2) in implementation and 17.4 (IQR: 16.5–18.3) in post-intervention. The COVID-19 period was associated with a substantial immediate decline in ALT testing (step −9.49, 95% CI −11.5, −7.52), followed by a transient increase (pulse 4.66, 95% CI 3.00, 6.32) and a modest positive monthly trend (ramp 0.27, 95% CI 0.01, 0.54). The result was a median testing rate of 16.1 (IQR: 14.7–17.6).

#### ALT plus AST

The median monthly rate of testing with ALT plus AST during pre-intervention was 1.09 (IQR 1.02–1.14) per 100 persons (Fig. 4C). Implementation of the policy was associated with a significant immediate increase in ALT plus AST testing (step: 0.16, 95% CI 0.02–0.30). Despite this, the median testing rate remained low during implementation (0.65, IQR: 0.62–0.70). In the post-intervention and COVID-19 periods, no significant step or ramp changes were observed. The median ALT plus AST monthly testing rate during these periods was 0.36 (IQR: 0.32–0.41) and 0.18 (IQR: 0.17–0.19) per 100 persons respectively.

## Discussion

In this large, population-based cohort of over 1.6 million adults with diabetes in Ontario, Canada, we observed shifts and persistent gaps in liver-related testing, marked by changes in public insurance coverage of AST. Despite an elevated risk of liver disease in this population, in any given year, approximately 39–47% were never assessed for liver damage with blood tests. Changes in testing practices were not aligned with evidence-based care. ALT testing alone was most common and steadily increased, whereas testing for FIB-4 components sharply declined following the implementation of reimbursement restrictions. These patterns were mirrored in interrupted time-series analysis, which demonstrated significant absolute reductions in monthly FIB-4 testing rates after implementation of coverage restrictions and during the COVID-19 pandemic. Together, our findings suggest that the attempt to reduce unnecessary AST testing may have unintentionally curtailed access to liver fibrosis-relevant testing amongst a high-risk group.

In the United Kingdom, only 1.5% of 26,090 people with diabetes received the tests necessary for calculating FIB-4 in primary or outpatient care over a 21-month period starting in December 2019 ^29^. We found higher proportions of people receiving the components of FIB-4 testing in Ontario, as the lowest proportion tested in our study was 6% during the COVID-19 period. Both our work and that in the United Kingdom were conducted when international associations recommended liver fibrosis screening for people with diabetes, while national groups in the respective countries lacked specific recommendations^29^. Factors associated with an increased risk of FIB-4 testing were similarly related to an increased likelihood of adherence to guidelines for screening for non-liver complications that can arise from diabetes in other countries, including female sex^30,31^, hypertension^31^, and older age^31,32^. There is established evidence on the relationship between factors such as female sex and increased healthcare utilization compared to males^33^. We report that people with less glycemic control were less likely to receive FIB-4 testing which aligned with the relationship observed with non-adherence to screening for complications such as chronic kidney disease or retinopathy^31,32,34^. We found no relationship between area-level income and the risk of FIB-4 testing which differed from two studies in the United States that modeled income at the area-level^32^ or with health insurance type as a proxy^31^. While we observed differences in testing patterns across patient characteristics, the primary objective of this study was to evaluate changes associated with the policy intervention. Therefore, these subgroup findings should be interpreted cautiously, as the study design does not allow causal inference regarding these factors.

Our results align with a study that examined all tests targeted by the first phase of Ontario’s Appropriateness Initiative, which found that the utilization of six of the eight tests significantly decreased from 2006 to 2018 in the general population^14^. AST utilization decreased by 21%, but this decrease was not found to be statistically significant^14^. Prior policies may have already reduced the use of AST. Particularly, the checkbox option was removed from the laboratory requisition forms in Ontario in 2007, hindering AST ordering convenience and resulting in a 46% reduction in testing^13^. In contrast, we observed an increase in ALT alone testing during the study period. ALT is conveniently available on requisition forms; this increase may reflect enhanced clinical monitoring. Evidence that HbA1c testing in the diabetes population in Ontario increased from 2005 to 2014 supports this hypothesis^35^. ALT is generally referred to as a ‘specific’ test because it is predominantly elevated in liver injury^36^. However, approximately 24–33% of people with advanced fibrosis have ALT levels that are considered normal^37,38^ and international guidelines do not consider ALT as an appropriate test to assess fibrosis stage.

Our analysis did not demonstrate an immediate reduction in FIB-4 testing following the 2013 policy change. One plausible explanation is that the financial implications of the AST restriction may not have been consistently enforced at the laboratory level immediately following policy introduction. Laboratories required specific wording to be included on the requisition form for AST testing to qualify for OHIP reimbursement; otherwise, the cost could be passed on to the patient. Evidence from laboratory communications suggests that education and clarification regarding how to complete requisition forms appropriately continued through March 2017^22^. This suggests that the policy’s operationalization, particularly the enforcement of patient charges, may have taken time to be implemented consistently. As enforcement and awareness increased over time, ordering behavior may have gradually adapted, contributing to the more pronounced declines observed in the post-implementation period.

Our results suggest that the AST reimbursement restriction in Ontario acts as a significant barrier to fibrosis screening by potentially discouraging PCPs from ordering appropriate tests. When AST is requested outside of OHIP coverage, patients may be more likely to opt out due to the cost. Given that Diabetes Canada has adopted a similar position on liver fibrosis screening as their international counterparts, advocating for the removal of the 2013 policy restriction is necessary but not sufficient. Barriers to implementing guidelines in primary care persist at multiple levels, relating to the provider (education), patient (motivations, expectations), institution (time constraints and lack of supports for primary care) or the guidelines themselves (inconsistencies)^39^. Efforts to increase compliance with clinical guidelines often involve multiple interventions and, increasingly, rely on multidisciplinary stakeholder engagement^40^.

This study has several important strengths. The longitudinal design—spanning more than a decade and over 4.7 million person-years of follow-up—enabled extensive evaluation of temporal trends and policy impacts across distinct eras. Moreover, the integration of system-, provider-, and patient-predictors allowed us to control for multilevel determinants of testing practices. We focused on a validated high-risk population, and by excluding individuals with pre-existing liver disease or elevated FIB-4 at baseline, those more likely to undergo routine liver risk assessment, enhanced the generalizability of our findings. The study also provides timely evidence, given the recent Diabetes Canada guidelines that recommend liver fibrosis risk assessment in this population.

Our study also has limitations. We assumed the testing modalities were intended to assess chronic liver damage. However, these tests may have been used for other purposes, such as to guide medication use – particularly statins – or to assess alcohol abuse. Additionally, there may have been a clinical indication for FIB-4 testing. Primary care pathways in Ontario suggest that in the presence of an incidental finding of steatosis, the result of a FIB-4 score can be used to determine whether referral to a liver specialist is necessary^41^. To focus on routine testing patterns, we censored individuals at the time of incident liver disease diagnoses, including MASLD and 15 other liver conditions identified using ICD codes. However, MASLD is frequently underdiagnosed and incompletely captured in administrative data, meaning some individuals with underlying disease may remain in the cohort. If these individuals undergo increased liver-related testing, this could modestly overestimate routine testing proportions; however, such misclassification is likely non-differential over time and therefore is unlikely to substantially affect the observed temporal trends of our ITS analysis. Additionally, PCPs may have used strategies to order AST (indicating it was on the advice of hepatology), which would have bypassed the patient paying out of pocket, reducing the impact of the policy. However, we do not anticipate that individual loopholes would significantly impact findings at the population level. We recognize that advocacy efforts by *Choosing Wisely Canada* – a campaign focused on the appropriate use of health interventions may have influenced AST testing practices concurrently with the OHIP restriction. Their impact is evident in the family medicine hospital clinics imposing their own interventions to reduce AST testing^42,43^ and in collaboration with the Canadian Society of Clinical Chemists, which defines ALT alone as the appropriate approach to liver screening^44^. Finally, international guidelines suggest screening people with type 2 diabetes. Administrative data do not reliably distinguish diabetes type and as a result, our cohort included all adults with diabetes. This approach maximizes sensitivity for capturing individuals with type 2 diabetes but reduces specificity, as a proportion of individuals with type 1 diabetes will also be included. Given that approximately 90–95% of diabetes in Ontario is type 2 diabetes^45^, the impact of this misclassification on the observed temporal trends is likely minimal and non-differential.

Despite increasing consensus on the importance of detecting advanced liver fibrosis in people with diabetes, we observed declines in FIB-4 testing. We highlight a persistent knowing–doing gap: evidence and international recommendations have not consistently translated into appropriate testing practices in Ontario. Policies originally designed to curb unnecessary testing have, over time, constrained access for a high-risk population. Resource stewardship remains an essential component of Canada’s healthcare landscape. Innovative solutions to balancing resource stewardship with evidence-based care can include modifying policy restrictions to focus on limiting AST testing to individuals with diabetes or adding a direct calculation for FIB-4 on requisition forms or electronic medical records to promote proper interpretation. However, our findings highlight the unintended consequences of cost-containment policies on evidence-based practice, which should be rectified as the burden of liver disease continues to grow worldwide.

## Supplementary Information

Below is the link to the electronic supplementary material.

Supplementary Material 1

## Data Availability

The dataset from this study is held securely in coded form at ICES. While legal data sharing agreements between ICES and data providers (e.g., healthcare organizations and government) prohibit ICES from making the dataset publicly available, access may be granted to those who meet pre-specified criteria for confidential access, available at www.ices.on.ca/DAS (email: das@ices.on.ca). The full dataset creation plan and underlying analytic code are available from the authors upon request, understanding that the computer programs may rely upon coding templates or macros that are unique to ICES and are therefore either inaccessible or may require modification.Any additional questions can be directed to the corresponding author.
