# Correction: Reimbursement restrictions reduce liver fibrosis screening in adults with diabetes in Ontario: a population-based interrupted time series analysis

**DOI:** 10.1038/s41598-026-64893-y

**Published:** 2026-08-04

**Authors:** Jessica Burnside, Maya Djerboua, Jennifer A. Flemming, Giada Sebastiani, William W. L. Wong, Zihang Lu, Harpreet S. Bajaj, Keyur Patel, Mia Biondi, Michael Betel, Amy Nahwegahbow, Zoë R. Greenwald, Erica E. M. Moodie, Sahar Saeed

**Affiliations:** 1 Department of Public Health Sciences, Queen’s University, 203 Carruthers Hall, 62 Fifth Field Company Lane, Kingston, ON K7L 3N6 Canada; 2 ICES Queen’s, Kingston, ON Canada; 3 Division of Gastroenterology and Hepatology, Department of Medicine, McGill University Health Centre, Montreal, QC Canada; 4 School of Pharmacy, University of Waterloo, Kitchener, ON Canada; 5 LMC Healthcare, Brampton, ON Canada; 6 Division of Gastroenterology and Hepatology, University Health Network Toronto, Toronto, ON Canada; 7 School of Nursing, York University, Toronto, ON Canada; 8 Fatty Liver Alliance, Toronto, ON Canada; 9 School of Public Health Sciences, University of Waterloo, Kitchener, Canada; 10 Dalla Lana School of Public Health, University of Toronto, Toronto, ON Canada; 11 Department of Epidemiology & Biostatistics, McGill University, Montreal, QC Canada

Correction to: *Scientific Reports* 10.1038/s41598-026-48600-5, published online 29 April 2026

The original version of this Article contained an error in the order of the panels in Figure 4, where panels A, B and C were published as panels C, A and B. The original Figure 4 and accompanying legend appear below.

**Fig. 4:**
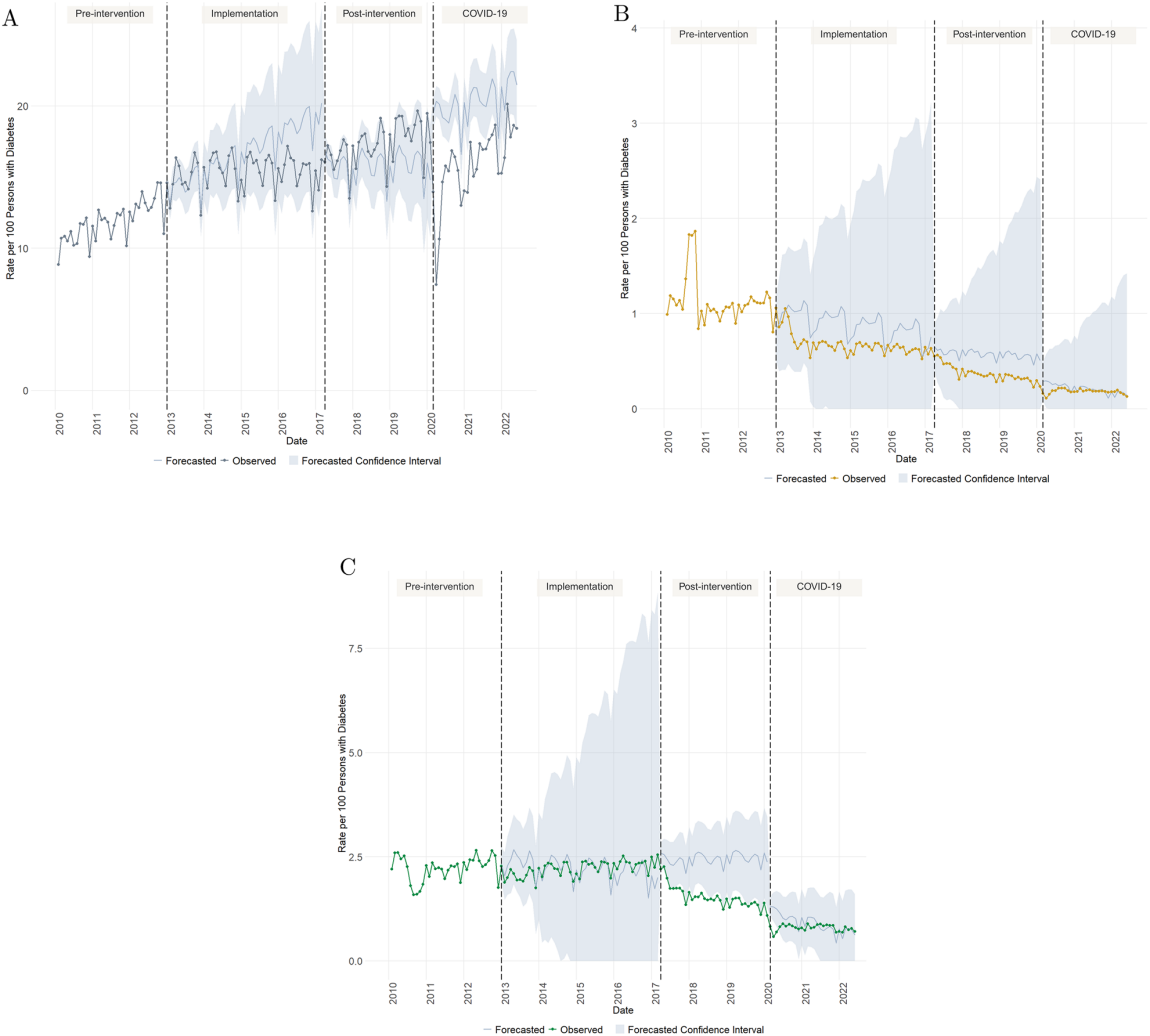
Impact of the policy periods on absolute testing rates. Line graphs show the observed monthly rate of testing with (**A**) FIB-4 (green), (**B**) ALT (grey), and (**C**) ALT plus AST (yellow). Forecasted rates calculated with the ARIMA models are depicted in light blue with shaded confidence intervals (cut-off at zero). Policy periods are delineated through the use of black vertical lines and beige labels at the top of the graph.

The original Article has been corrected.

