# Supplementary material for: Reimbursement restrictions reduce liver fibrosis screening in adults with diabetes in Ontario: a population-based interrupted time series analysis
Source: Sci Rep. 2026 Apr 29;16:19842. doi: 10.1038/s41598-026-48600-5 (PMC13315214; doi:10.1038/s41598-026-48600-5)
Supplement: Supplementary file 1 — Supplementary Material 1 [file 41598_2026_48600_MOESM1_ESM.docx]

**Supplemental 1. Description of ICES Databases Analyzed**

The Discharge Abstract Database, National Ambulatory Care Reporting System, and OHIP Claims Database provided hospitalization, emergency department visit, and physician claim data, respectively, to identify diagnoses of liver conditions and relevant hospital stays or procedures. Laboratory testing information was collected from the Ontario Laboratories Information System (OLIS). The Registered Persons Database was used to obtain sociodemographic information. Area-level proxies for socioeconomic status were obtained from the Ontario Marginalization Index and the Statistics Canada Postal Code Conversion File. The Same Day Surgery database was used to indicate morbidity and healthcare system use. The Ontario Hypertension Dataset was used to identify individuals with hypertension. The Assistive Devices Program was used to apply a validated algorithm for type 1 diabetes. Pregnant people were determined using the MOMBABY database. Provider information was sourced from the Estimated Schedule of Benefits price associated with each OHIP fee code and suffix in the database, as well as the Client Agency Program Enrolment database. External data from Public Health Ontario Laboratory was linked to ICES to identify individuals with hepatitis B or C.

**Supplemental 2. International Classification of Diseases for a) liver-related conditions and b) other covariates**

|  | **Data Source** | | | |
| --- | --- | --- | --- | --- |
|  | **DAD or NACRS** | | **OHIP** | |
| **Condition** | **ICD-9 codes** | **ICD-10-CA Codes** | **ICD-9 (3-digit) codes** | **Fee Codes** |
| 1. **Liver Conditions** | | | | |
| Alcohol-associated liver disease | 571.0, 571.1, 571.2, 571.3 | K70 |  |  |
| Alpha-1-antitrypsin deficiency | 273.8 | E88.0A, E88.0B |  |  |
| Autoimmune liver disease (includes autoimmune hepatitis, primary biliary cholangitis, primary sclerosing cholangitis) | 571.6, 576.1 | K83.0A, K83.0F, K74.3, K75.4 |  |  |
| Budd-Chiari syndrome | 453.0, 573.8 | I82.0, K76.5 |  |  |
| Chronic hepatitis, unspecified | 571.4 | K73.9, K73.2 |  |  |
| Cirrhosis (includes compensated cirrhosis or esophageal/gastric varices, not bleeding) | 571.2, 571.5, 456.1 | K70.3, K71.7, K74.6, I85.9, I98.2, I98.21 | 571 |  |
| Chronic liver disease | 070.2, 070.3, 070.4, 070.5, 070.6, 070.9, 571.0, 571.1, 571.2, 571.3, 571.4, 571.8, 571.5, 571.6, 571.9, 570, 572.2, 572.8, 573.1, 573.3, 572.3, 572.4, 573.0, 573.4, 573.8, 573.9, 573.2, 456.0, 456.1, 456.2, 782.4, 789.5, 789.1, 275.0, 275.1 | B16, B17, B18, B19, I85, I98.2, I98.3, R17, R18, R16.0, R16.2, B94.2, Z22.5, E83.1, E83.0, K70, K71.3, K71.4, K71.5, K71.7, K72.1, K72.9, K73, K74, K75.3, K75.4, K75.8, K75.9, K76, K77 | 571, 573, 070 | Z551, Z554 (Liver incision biopsy) |
| Hemochromatosis | 275.0 | E83.1 |  |  |
| Liver malignancy (including HCC and IHCC) | 155 | C22 | 155 |  |
| Liver transplant^a^ |  |  |  | S294, S266 |
| Secondary or unspecified biliary cirrhosis | 571.6 | K74.4, K74.5 |  |  |
| MASLD/NAFLD | 571.8 | K76.0 |  |  |
| MASH/NASH | 573.3 | K75.8 |  |  |
| Wilson’s disease | 275.1 | E83.0B |  |  |
| 1. **Covariates** | | | | |
| Alcohol use disorder | 291, 303, 305.0 | F10 | 291, 303 |  |
| Substance use disorder (except for alcohol and nicotine/caffeine) | 304.0, 304.7, 305.5, 304.3, 305.2, 304.1, 305.4, 304.2, 305.6, 304.5, 305.3, 304.6, 292.0, 292.1, 292.2, 292.8, 292.9, 304.8, 304.9, 305.8, 305.9 | F11-F14, F16, F18, F19 | 292, 304 |  |

Note that hepatitis B or C are omitted from this table because they were identified using Public Health Ontario indicator flag. Prevalent liver diseases were identified from the earliest date of data availability: 1991 for the OHIP Claims Database and 1988 for the Discharge Abstract Database.

^a^Liver transplant was identified using Canadian Classification of Health Interventions (1OA85) and the older version of these, the Canadian Classification of Diagnostic, Therapeutic, and Surgical Procedures (6240, 6241, 6249)

**Supplemental 3. LOINCs used to identify laboratory tests**

| **Laboratory Test** | **LOINCs** |
| --- | --- |
| CBC Hemoglobin | 20509-6, 20570-8, 4544-3, 718-7, 71833-8 |
| HbA1c | 17855-8, 17856-6, 41995-2, 4548-4, 59261-8, 71875-9 |
| Serum creatinine | 14682-9 |
| Glucose | 14749-6, 14752-0, 14753-8, 14754-6, 14756-1, 14757-9, 14759-5, 14761-1, 14763-7, 14764-5, 14765-2, 14766-0, 14767-8, 14771-0, 14995-5, 14996-3, 15074-8, 1552-9, 20441-2, 25663-6, 25665-1, 25668-5, 25673-5, 25677-6, 25680-0, 32319-6, 32320-4, 32321-2, 34059-6, 39480-9, 39481-7, 40148-9, 40150-5, 40162-0, 40193-5, 40287-5, 4269-7, 45055-1, 47622-6, 51596-5, 51597-3, 53094-9, 55351-1, 55381-8, 59791-4, 59792-2, XON10304-4, XON10395-2, XON10432-3, XON10433-1, XON11912-3, XON12555-9 |
| Sodium | 2951-2 |
| Any of the liver fibrosis tests (ALT, AST, platelets) | 1920-8, 1742-6, 1743-4, 1744-2, 28542-9, 32623-1, 777-3 |

**Supplemental 4. Covariate Definition Details**

Demographics included sex and age (categorized 40-49 years, 50-59, 60-69, 70-79, and 80+). International Classification of Diseases, ninth (ICD-9) and tenth (ICD-10) revisions, were used to define alcohol and substance use disorder in hospitalizations, emergency departments, and physician claims (Appendix 2). Features of diabetes included hemoglobin A1c levels [(HbA1c) categorized <7%, 7-8.5% and >8.5% (1)], and disease duration calculated using diagnosis date (categorized <2 years, 2-5, 6-10, 11-15, 16-20, 20+). Type 1 diabetes was identified using 2 validated algorithms: one with the highest positive predictive value and the other with the highest sensitivity for administrative data (2). A validated algorithm was used to identify hypertension, with sensitivity 73%, specificity 95% (3). Aggregated diagnosis groups and resource utilization bands, assessed using The John Hopkins ACG^®^ System (4), were interpreted as indicators of morbidity and system use, respectively. Rurality was defined using a threshold of community size ≤10,000 people. Area-level deprivation was measured using i) age and labor force, ii) material resources, iii) racialized and newcomer populations, and iv) household and dwellings quintiles (5). Income was derived from area-level average household income after-tax collected during the census of population every 5 years and categorized in quintiles (6). Provider-level information was used to ascertain whether an individual was rostered with primary care, virtually or otherwise.

References:

1. Imran SA, Agarwal G, Bajaj HS, Ross S. Targets for Glycemic Control. Canadian Journal of Diabetes. 2018;42:S42-S6.

2. Weisman A, Tu K, Young J, Kumar M, Austin PC, Jaakkimainen L, et al. Validation of a type 1 diabetes algorithm using electronic medical records and administrative healthcare data to study the population incidence and prevalence of type 1 diabetes in Ontario, Canada. BMJ Open Diabetes Res Care. 2020;8(1).

3. Tu K, Campbell NR, Chen ZL, Cauch-Dudek KJ, McAlister FA. Accuracy of administrative databases in identifying patients with hypertension. Open Med. 2007;1(1):e18-26.

4. The John Hopkins ACG® System. Excerpt from Version 11.0 Technical Reference Guide. 2014 [Available from: https://www2.gov.bc.ca/assets/gov/health/conducting-health-research/data-access/johns-hopkins-acg-system-technical-reference-guide.pdf.

5. Public Health Ontario. Ontario Marginalization Index (ON-Marg). [Available from: https://www.publichealthontario.ca/en/Data-and-Analysis/Health-Equity/Ontario-Marginalization-Index.

6. Canadian Institute for Health Information. Measuring Health Inequalities: A Toolkit. Area-Level Wquity Stratifiers Using PCCF and PCCF+. 2018 [Available from: https://www.cihi.ca/sites/default/files/document/toolkit-area-level-measurement-pccf-en.pdf.

**Supplemental 5. Hypothetical illustration of at-risk time for 3 individuals for the first 3 years of the study period**


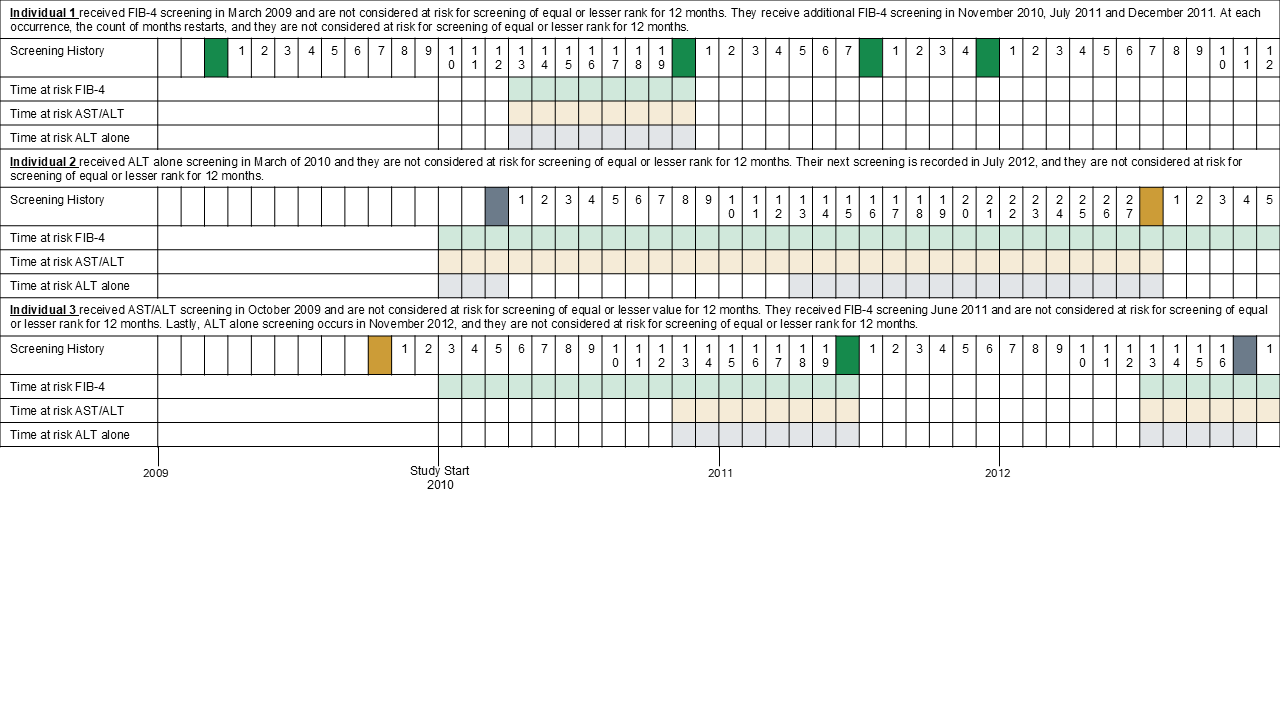
Each box represents a month. The color in the ‘Screening History’ rows indicate the screening type (green = FIB-4; yellow = ALT plus AST; grey = ALT only). The number of months since screening are counted in the ‘Screening History’ row. Shaded boxes in the ‘Time at risk’ rows indicate months when the individual is considered at risk.

**Supplemental 6. The proportion of people with diabetes that received each screening modality for the 4 policy eras.**

**
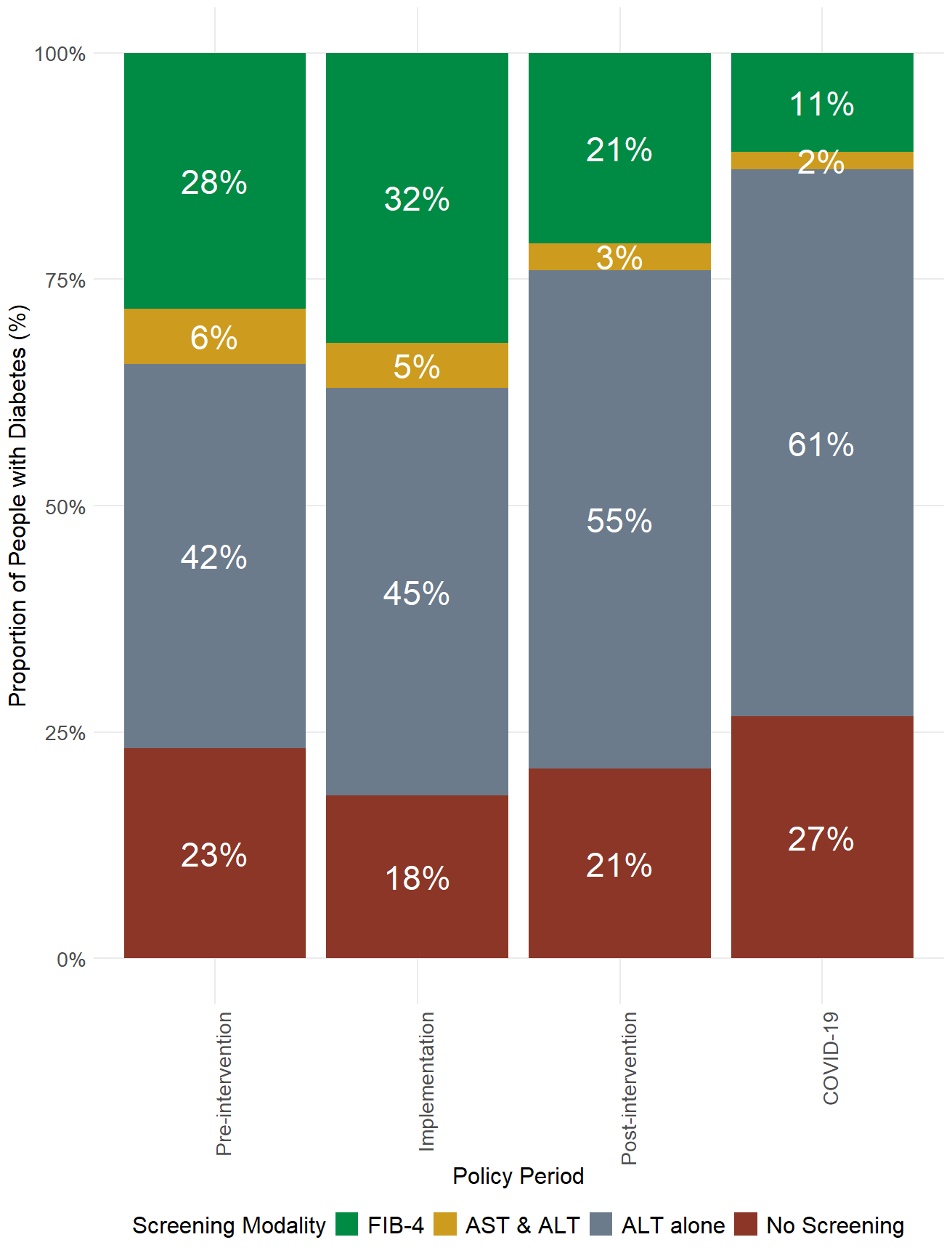
**

**Supplemental 7. Rate changes according to step, ramp, and pulse functions from ARIMA models.**

| **FIB-4** | | | |
| --- | --- | --- | --- |
| **Policy Era** | **Interpretation** | **Estimate (95%CI)** | **p-value** |
| Implementation | Immediate and sustained change in monthly screening rate | 0.07 (-0.14, 0.28) | 0.52 |
|  | Monthly rate change | 0.004 (-0.04, 0.05) | 0.87 |
| Post-intervention | Immediate and sustained change in monthly screening rate | -0.38 (-0.59, -0.18) | 0.0003* |
|  | Monthly rate change | -0.03 (-0.07, 0.01) | 0.19 |
| COVID-19 | Immediate and sustained change in monthly screening rate | -0.70 (-1.00, -0.41) | <0.0001* |
|  | Immediate and transient change in monthly screening rate | 0.25 (0.04, 0.46) | 0.02* |
|  | Monthly rate change | 0.02 (-0.02, 0.07) | 0.29 |
| **ALT plus AST** | | | |
| **Policy Era** | **Interpretation** | **Estimate (95%CI)** | **p-value** |
| Implementation | Immediate and sustained change in monthly screening rate | 0.16 (0.02, 0.30) | 0.03* |
|  | Monthly rate change | -0.02 (-0.06, 0.02) | 0.40 |
| Post-intervention | Immediate but sustained change in monthly screening rate | -0.11 (-0.25, -0.04) | 0.14 |
|  | Monthly rate change | -0.01 (-0.05, 0.03) | 0.69 |
| COVID-19 | Immediate but sustained change in monthly screening rate | -0.13 (-0.32, -0.07) | 0.19 |
|  | Immediate and transient change in monthly screening rate | 0.03 (-0.11, 0.17) | 0.66 |
|  | Monthly rate change | 0.01 (-0.03, 0.05) | 0.65 |
| **ALT** | | | |
| **Policy Era** | **Interpretation** | **Estimate (95%CI)** | **p-value** |
| Implementation | Immediate and sustained change in monthly screening rate | 0.54 (-1.01, 2.09) | 0.49 |
|  | Monthly rate change | -0.09 (-0.33, 0.16) | 0.50 |
| Post-intervention | Immediate and sustained change in monthly screening rate | 0.07 (-1.47, 1.62) | 0.93 |
|  | Monthly rate change | 0.08 (-0.17, 0.32) | 0.55 |
| COVID-19 | Immediate and sustained change in monthly screening rate | -9.49 (-11.5, -7.52) | <0.0001* |
|  | Immediate and transient change in monthly screening rate | 4.66 (3.00, 6.32) | <0.0001* |
|  | Monthly rate change | 0.27 (0.01, 0.54) | 0.04* |
